# Vascularisation is not necessary for gut colonisation by enteric neural crest cells^☆^

**DOI:** 10.1016/j.ydbio.2013.11.007

**Published:** 2014-01-15

**Authors:** Jean-Marie Delalande, Dipa Natarajan, Bertrand Vernay, Malcolm Finlay, Christiana Ruhrberg, Nikhil Thapar, Alan J. Burns

**Affiliations:** a Neural Development Unit, UCL Institute of Child Health, 30 Guilford Street, London WC1N 1EH, United Kingdom; b UCL Institute of Ophthalmology, 11-43 Bath Street, London EC1V 9EL, United Kingdom; c Department of Clinical Genetics, The Erasmus University Medical Center, Rotterdam, The Netherlands

**Keywords:** Enteric nervous system, Vascular system, Neural crest cells, Blood vessels, Migration

## Abstract

The vasculature and nervous system share striking similarities in their networked, tree-like architecture and in the way they are super-imposed in mature organs. It has previously been suggested that the intestinal microvasculature network directs the migration of enteric neural crest cells (ENCC) along the gut to promote the formation of the enteric nervous system (ENS). To investigate the inter-relationship of migrating ENCC, ENS formation and gut vascular development we combined fate-mapping of ENCC with immunolabelling and intravascular dye injection to visualise nascent blood vessel networks. We found that the enteric and vascular networks initially had very distinct patterns of development. In the foregut, ENCC migrated through areas devoid of established vascular networks. In vessel-rich areas, such as the midgut and hindgut, the distribution of migrating ENCC did not support the idea that these cells followed a pre-established vascular network. Moreover, when gut vascular development was impaired, either genetically in *Vegfa*^*120/120*^ or *Tie2-Cre;Nrp1*^*fl*/−^ mice or using an *in vitro Wnt1-Cre;Rosa26*^*Yfp/+*^ mouse model of ENS development, ENCC still colonised the entire length of the gut, including the terminal hindgut. These results demonstrate that blood vessel networks are not necessary to guide migrating ENCC during ENS development. Conversely, in *miRet*^*51*^ mice, which lack ENS in the hindgut, the vascular network in this region appeared to be normal suggesting that in early development both networks form independently of each other.

## Highlights

• The gut vasculature is thought to direct migrating enteric neural crest cells (ENCC).
• We visualised ENCC and the vasculature in the developing gastrointestinal tract.
• Enteric and vascular networks initially had little correlation.
• ENCC colonised the gut when vascular development was impaired *in vivo* or *in vitro.*
• Blood vessels are thus not necessary to guide migrating ENCC during ENS formation.

## Introduction

During vertebrate development, organs and tissues must connect to the blood vascular system to receive fluids, nutrients and oxygen, and to the nervous system to receive and send sensory, autonomic or functional information. Consequently, both mature networks share obvious similarities at the anatomical level, and they also use similar cellular and molecular mechanisms to orchestrate their parallel developmental programs (Bates et al., 2003, Eichmann et al., 2005, Eichmann and Thomas, 2013, Tam and Watts, 2010). Recent evidence suggests that the two networks also influence each other's development through direct molecular interactions. For example, neuronal progenitors and neurons secrete the vascular growth factor VEGF to stimulate or pattern their vascular supply (Haigh et al., 2003, Mukouyama et al., 2002, Raab et al., 2004, Rosenstein et al., 2010, Ruhrberg, 2003), and vessels, comprised of endothelial cells (EC) and mural cells, release neurotrophic factors such as artemin and neurotrophin-3 to attract nerve fibres (Honma et al., 2002, Kuruvilla et al., 2004, Weinstein, 2005). Additionally, the alpha-chemokine receptor CXCR4 and its ligand SDF1, which are essential for gastrointestinal tract vascularisation (Tachibana et al., 1998), also promote the migration of cranial neural crest cells (NCC) (Rezzoug et al., 2011, Theveneau et al., 2010).

Although the nervous system of the gut contains extrinsic components, its main functional unit is the intrinsic enteric nervous system (ENS). The ENS consists of networks of interconnected ganglia embedded in the wall of the digestive tract and is the largest division of the peripheral nervous system (Furness, 2012, Gershon, 1999). The ENS is derived from NCC, which delaminate and migrate extensively to colonise the entire length of the gut (Goldstein et al., 2013, Laranjeira and Pachnis, 2009, Obermayr et al., 2013, Sasselli et al., 2012). Concomitant with the migration of ENCC within the gut is the development of the gut vascular system, which has not been as extensively investigated. Studies using Tg(tie1:H2B-eYFP) quail embryos (Sato et al., 2010), in which EC express YFP, revealed the presence of scattered EC within the gut mesenchyme as early as embryonic day (E)3 (Thomason et al., 2012). These cells become organised into a honeycomb pattern by E6, and by E10 are reshaped into a branching vascular pattern that extends from the mesentery. Additionally, studies in mice have shown that cells from the serosal mesothelium undergo EMT to join the blood vessels, where they give rise to the mural cells (Wilm et al., 2005). Eventually, the digestive tract receives blood supply from three branches of the abdominal aorta: the coeliac, superior and inferior mesenteric arties that supply the foregut, midgut and hindgut respectively (Geboes et al., 2001, Tachibana et al., 1998). In the functional adult gut, recent studies have revealed close anatomical associations between the ENS and capillaries that are specific to the villi, the crypts and the myenteric plexus (Fu et al., 2013). One possibility is that this co-patterning in the mature gut arises from co-dependent interactions during early development. Indeed, recent studies in quail suggested that the pre-established hindgut microvasculature serves a critical role in promoting and directing ENCC migration along the hindgut (Nagy et al., 2009). Moreover, the intestinal vascular mesentery has been proposed to serve as an important migratory route for ENCC to colonise the terminal hindgut in mice (Nishiyama et al., 2012). Thus the aim of this study was to investigate the potential inter-relationship between the developing ENS and the gut vasculature.

We used chick^GFP^–chick intraspecies grafting to permanently label and fate-map vagal ENCC with GFP, combined with intravascular injection of the lipophilic dye DiI to stain the nascent blood vessel network. Because DiI highlighted only patent blood vessels, we also labelled quail embryos with QH1 and HNK1 antibodies to mark endothelial and neural crest-derived networks, respectively. The analysis of wholemount preparations by high magnification confocal imaging showed little correlation between migrating NCC and blood vessel development. Consistent with these observations we showed, using mouse models defective in vascular development, and an *in vitro* assay, that ENCC can colonise the entire length of the gut independently of the vascular network.

## Materials and methods

### Chick^GFP^–chick intraspecies tissue grafting and blood vessel labelling with DiI injection

**Fig. 1:**
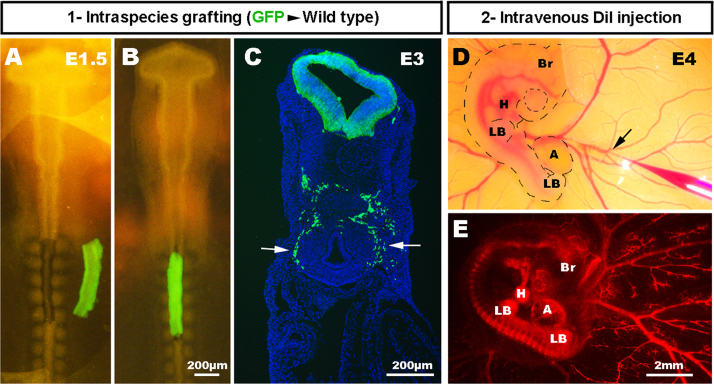
Labelling of the chick enteric nervous and vascular systems. In order to label ENCC and blood vessels, the vagal region of the neural tube is surgically removed from E1.5 (HH10) wild type host chicken embryos (A) and replaced with the equivalent region of neural tube from a GFP+ donor chicken (B). All vagal-derived ENCC are therefore GFP+ [arrows] (C). In the same neural tube-transplanted embryo at E4 (HH23), DiI is injected into the blood stream [black arrow] (D), to reveal the developing blood vessel network (E) Br: Brain; H: Heart; LB: limb bud; A: Allantois. Scale bar A and B=200 μm; C=200 μm; D and E=2 mm.

Fertile chicken eggs, obtained from commercial sources, and transgenic GFP chicken eggs, obtained from The Roslin Institute, The University of Edinburgh (McGrew et al., 2004), were incubated at 37 °C and staged according to the embryonic day of development (E), and by using the developmental tables of Hamburger and Hamilton (1951). For chick^GFP^–chick grafting, the neural tube and associated neural crest, adjacent to somites 2–6 inclusive, was microsurgically removed from normal chick embryos at embryonic day E1.5 and replaced with equivalent stage-matched tissue obtained from chick^GFP^ embryos (Fig. 1), as previously described (Burns and Le Douarin, 1998, Freem et al., 2012) Following grafting, eggs were returned to the incubator, and embryos allowed to develop up to a further 10 days such that GFP+NCC colonised the gut and lungs. For DiI injection in chick embryos at stages E4.5–E11.5, CellTracker^TM^ CM-DiI (Molecular Probes^®^) was injected into the peripheral vascular network using a fine glass needle connected to a mouth pipette (Fig. 1). The dye was injected slowly and allowed to diffuse within the blood flow for two to three minutes to ensure staining of the entire lumenised blood vessel network to its finest capillaries. The labelled embryos were harvested and fixed in 4% paraformaldehyde (PFA) shortly after DiI injection and mounted under a bridged coverslip using Vectashield mounting medium (Vector Laboratories).

### Mouse strains

To obtain mouse embryos of defined gestational ages, mice were mated in the evening, and the morning of vaginal plug formation was counted as 0.5 days post coitum (dpc). Mice carrying the *Vegfa*^*120*^ mutation (MGI:1931047) (Carmeliet et al., 1999) have been described (Ruhrberg et al., 2002). Conditional null mutants for *Nrp1* (*Nrp1*^*fl/f)*^*)* (MGI:3512103) (Gu et al., 2003) were mated to mice expressing Cre recombinase under the control of the endothelial-specific *Tie2* promoter (MGI:2450311) (Kisanuki et al., 2001). Mice carrying a conditional *Yfp* allele in the Rosa26 locus (MGI:2449038) (Srinivas et al., 2001) were mated to mice expressing Cre recombinase under the control of the Wnt1 promoter/enhancer (MGI:2386570) (Danielian et al., 1998). Mice carrying the *miRet*^*51*^ mutation (MGI:2387446) have been described (de Graaff et al., 2001). Genotyping protocols can be supplied on request. Mouse husbandry was performed in accordance with UK Home Office and Institutional guidelines.

### Culture of foetal mouse intestine

Foetal mouse intestines were cultured *in vitro* as previously described (Natarajan et al., 1999). Briefly, transgenic *Wnt1-Cre;R26R*^*yfp/+*^ intestines with the mesentery attached were dissected from E11.5 embryos and placed in culture with OptiMEM (Invitrogen, UK) supplemented with 1 mM l-Glutamine (Invitrogen, UK) and 1 mM Penicillin/Streptomycin antibiotic mixture (Invitrogen, UK). Gastrointestinal tracts were cultured up to 4 days then fixed in 4% PFA for 1 h and processed for immunofluorescence labelling as described below.

### Immunofluorescence

For wholemount immunofluorescence labelling of quail (obtained from commercial sources) and mouse tissues, dissected gastrointestinal tracts were fixed for 1–2 h in 4% PFA in PBS then rinsed 3 times in PBS at room temperature and processed as previously described (Freem et al., 2010). Briefly, antibody blocking solution (10% sheep serum, 1% Triton-X-100 in PBS) was applied for 1 h at room temperature then samples were rinsed extensively in PBS and incubated in endomucin (Santa Cruz) and TuJ1 (Covance) primary antibodies diluted in antibody blocking solution overnight at 4 °C. Samples were then washed three times in PBS for 20 min and incubated with fluorescently tagged secondary antibodies (anti-rat Alexa568 and anti-mouse Alexa488, respectively) for 4 h at room temperature. Samples were washed for 1 h, and stained for 10 min with DAPI, before being mounted under a coverslip using Vectashield mounting medium (Vector Laboratories). Images were acquired with a Zeiss Axioskop fluorescent microscope or a Zeiss LSM 710 confocal microscope. For counting ENS cells in mouse tissues, sequential z-stack images were analysed, and TuJ1/DAPI+ cells counted using the Image-J Fiji cell counter plugin. Once imaged using confocal microscopy, the same samples were un-mounted, then cryoprotected in 15% sucrose in PBS, and put in gelatin blocks as previously described (Barlow et al., 2008). Frozen sections were cut at 12 μm using a Leica CM1900 cryostat at −22 °C. In the case of the foetal mouse intestine explants, the sections we re-immunostained using PECAM/CD31 (BD Pharmingen™). Imaging was carried out on a Zeiss Axioskop fluorescent microscope as above.

### 3D reconstruction

Confocal Z-stacks of chicken tissues from GFP-WT chimera injected with DiI, or from quail and mouse intestine double immunostained for endomucin and TuJ1, acquired with the Zeiss LSM 710, were reconstructed with the 3D viewer in Fiji ImageJ (Schindelin et al., 2012). Images were rotated 90° around the *X* axis for ZX orthogonal projections and the *Y* axis for YZ projections.

## Results

### Early migrating ENCC colonise gut regions devoid of vasculature

**Fig. 2:**
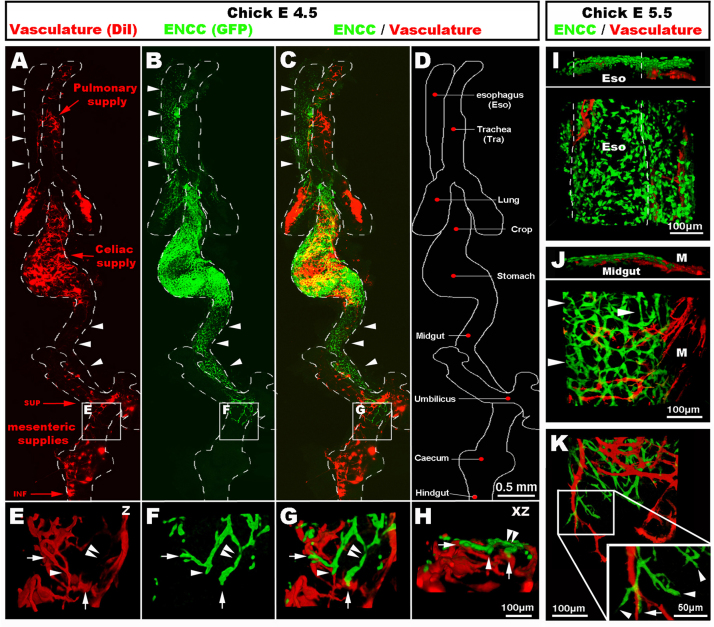
Wholemount preparation of chick gut and respiratory system at E4.5 (HH24) and E5.5 (HH28), showing distinct patterns of ENS and vascular network development. (A and C) The vascular networks develop from the pulmonary supply to the lungs, and from the coeliac supply to the stomach and the superior and inferior mesenteric supplies to the distal gut. The oesophagus and ventral midgut are devoid of blood vessels at E4.5 (arrowheads). (B and C) ENCC colonise the gut in a proximo-distal direction. ENCC migrate along and within the oesophagus and midgut despite the absence of vasculature (arrowheads). (E–H) High magnification images of the migration front (boxed in A–C) in the vascular rich region of the umbilicus. ENCC are both in the interspace between vessels (arrowhead) and in close association with blood vessels (arrows). (I–K) High magnification images at E5.5 (HH28). (I) The oesophagus is avascular yet ENCC formed an ENS. (J) ENCC have colonised the majority of the midgut including regions devoid of capillaries (J, arrowheads). (K) At the migration front, most mesenteric projections are perpendicular to the ENCC path. ENCC are found both in the interspace between vessels (arrowhead) and in close association with blood vessels (arrows). Z: z stack projection 3D reconstruction. XZ: 90° rotation around the X axis. Scale bar A–D=0.5 mm; E–H=100 μm; I–K=100 μm.

**Fig. 3:**
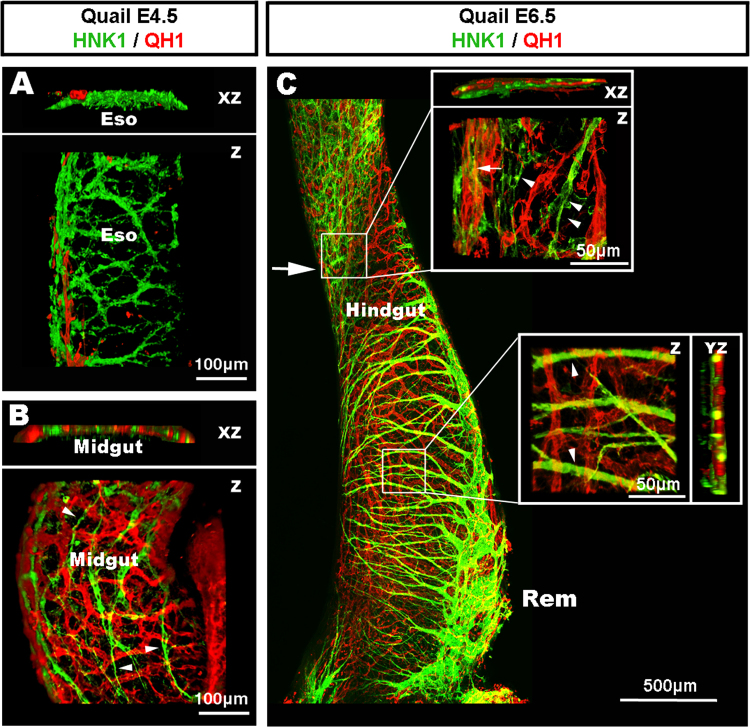
Formation of the enteric nervous and vascular systems in the embryonic quail digestive tract. (A) Confocal image of a wholemount preparation of oesophagus at E4.5 (quail stage Q25), labelled with QH1 and HNK1, showing extensive colonisation of the oesophagus by ENCC despite the lack of vasculature. (B) Confocal image of a wholemount preparation of midgut at E4.5 (Q25), labelled with QH1 and HNK1, at the level of the ENCC migration front. XZ projection reveals the localisation of both networks within the same layer of the midgut. Despite this co-localisation, the migration paths of the ENCC do not systematically follow the honeycomb pattern of the QH1+ network (arrowheads). (C) Confocal image of a wholemount preparation of hindgut at E6.5 (Q30), showing the ENCC migration front half way along the hindgut (arrow). Upper box: high magnification image in the region of the migration front reveals a lack of co-patterning between the ENCC and the QH1+ cell network. Both networks are sometimes closely co-localised (arrow) and sometimes apart (arrowheads). Lower box: high magnification demonstrating a lack of co-patterning between the projections of the nerve of Remak and the QH1+ cell network (arrowheads). Rem: Remak nerve. Z: z stack projection 3D reconstruction. XZ: 90° rotation around the *X* axis. Scale bar A and B=100 μm; C=500 μm.

**Fig. S1:**
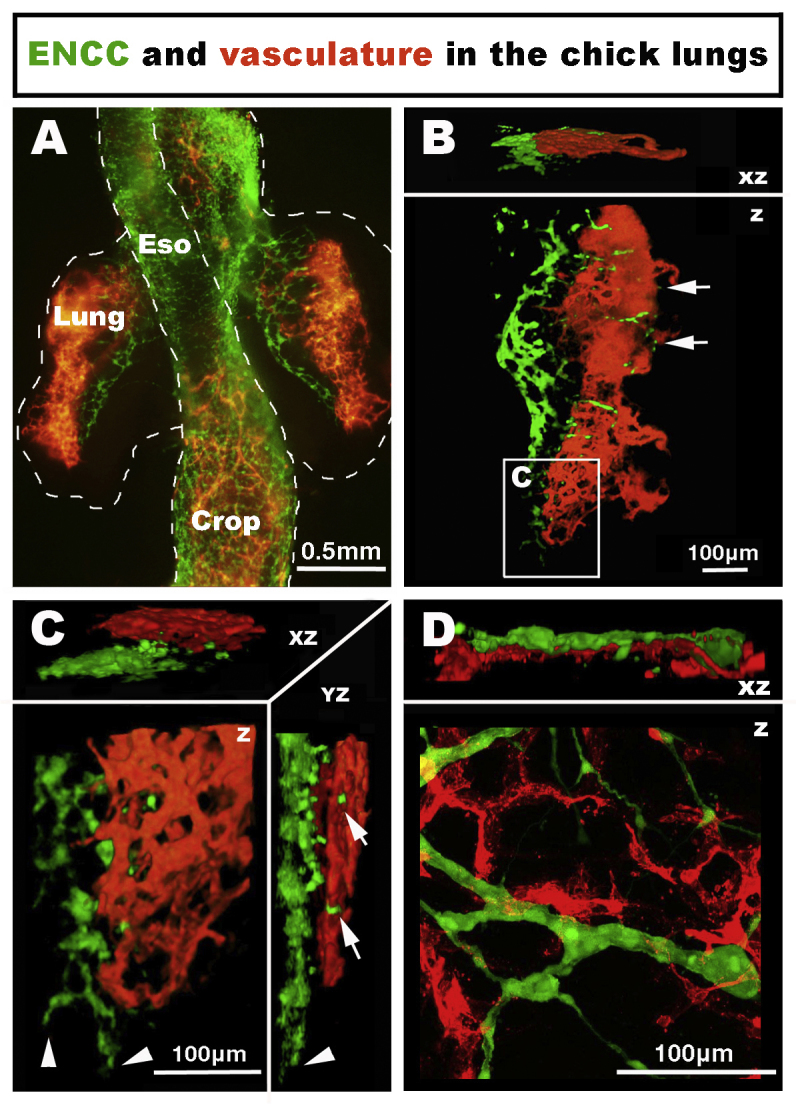
Formation of the neural crest-derived nervous and vascular systems in the embryonic chicken lungs. (A–C) Wholemount preparation of the lungs at E5.5 (HH28). NCC are located within the medio-dorsal region of the lungs, whereas the vascular system is located on the lateral side. In the most distal portion of the lungs, the NCC migration front is advanced more distally than the vascular network (C, arrowheads). Some isolated NCC are associated with the vascular system (B and C; arrows). (D) High magnification confocal image of the lungs at E11.5 (HH37-38). NCC form a thin ganglionated network intertwined with the vascular system.

**Fig. S2:**
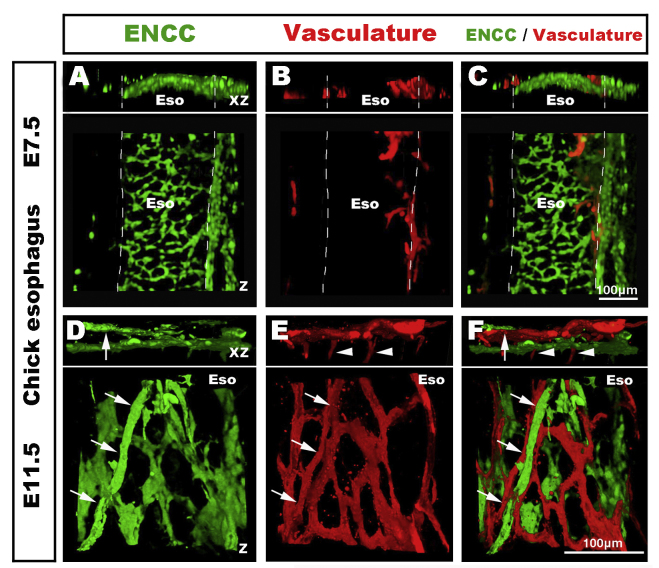
Formation of the enteric nervous and vascular systems in embryonic chicken oesophagus at E7.5 and E11.5. (A–C) Confocal imaging of the oesophagus at E7.5 (HH32) reveals very sparse vasculature limited to isolated vessels from the adjacent mesentery. (D–E) Confocal imaging of the oesophagus at E11.5 (HH37-38). The vascular system is present on the periphery of the oesophagus, separate from the ENS, and centripetal vessels extend across the ENS (E and F; arrowheads). Congruence is observed between blood vessels and extrinsic vagus nerve fibres (D and F; arrows).

Following intraspecies grafting and DiI intra-vascular injection, wholemount preparations of the gastrointestinal (GI) tract and respiratory system at E4.5 (HH24) showed the developing ENS labelled in green with GFP, because vagal ENCC are derived from the GFP graft (Fig. 1A–C; Fig. 2B and C), and the vascular system labelled in red by DiI (Fig. 1D, E and Fig. 2A, C). The two networks showed distinct patterns of development. As seen in these preparations, and as previously described (Burns and Le Douarin, 2001, Freem et al., 2012), vagal ENCC migrate in a proximo-distal direction, colonising first the oesophagus and progressing distally to eventually reach the terminal hindgut. At E4.5, the migration front had reached the umbilicus, but the distal midgut and the hindgut were un-colonised (Fig. 2B and C). The gut vascular network, on the other hand, does not form in a proximo-distal gradient, but from the three entry points of the coeliac supply, and the superior and inferior mesenteric arteries along the gut (Fig. 2A). This early pattern of GI vascular development initially leaves many regions, such as the oesophagus and the ventral part of the proximal midgut, free from a vascular network at stages when these regions have already been colonised by migrating ENCC (Fig. 2A–C and I). Confocal images of the migration front in vascularised regions of the midgut showed that migrating ENCC did not necessarily match the blood vessel layout, as migrating chains were often located in the interspace between blood vessels (Fig. 2E–H and K). At the migration front, some of the leading ENCC made close contact with the vasculature (Fig. 2E–H and K, arrows), whereas others did not (Fig. 2E–H and K, arrowheads) (also see Supplementary movie S1). Moreover, the midgut vasculature developed from mesenteric projections, which were mainly orientated perpendicular to the ENCC migration path (Fig. 2J and K). As these experiments in chick specifically examined the relationship between patent blood vessels (as shown by DiI labelling) and ENCC, we also utilised quail embryos to take advantage of the quail-specific EC maker QH1, and HNK1 to label neural crest-derived cells. In wholemount preparations at E4.5, we again found that the oesophagus was mostly avascular and lacking in EC, whereas an extensive network of HNK-1+ cells was present (Fig. 3A). At this stage, the migration front of ENCC was located proximal to the umbilicus. As previously described (Nagy et al., 2009), XZ projections of confocal images in this region showed that ENCC were migrating adjacent to the QH1+ cells in the presumptive myenteric region (Fig. 3B). However, the migrating path of HNK-1+ ENCC did not match the honeycomb pattern of the QH1+ cells, which were, as seen in chick, often orientated perpendicular to the ENCC migration path (Fig. 3B; arrowheads). Together, observations of wholemount preparations of the digestive and respiratory (Fig. S1) tracts showed little correlation between the nascent vascular system and the NCC migration paths. Amongst the various regions that exemplified this lack of correlation, the oesophagus was the most striking because wholemount preparations at E4.5 and E5.5 (Fig. 2) and later developmental stages (E7.5–E11.5; Fig. S2) showed that the oesophagus was mostly avascular during early development, but fully colonised by ENCC. These observations clearly demonstrate the ability of the ENCC to colonise an organ prior to its vascularisation.

Supplementary materials related to this article can be found online at doi:10.1016/j.ydbio.2013.11.007.

The following is the Supplementary material related to this article Video 1.

Video 1 3-dimensional rotation of the image in Fig. 2H, showing the vascular system and the ENCC migration front in the region of the umbilicus at E4.5 (HH24).

### The pre-established hindgut vasculature does not dictate the paths of migrating ENCC

**Fig. 4:**
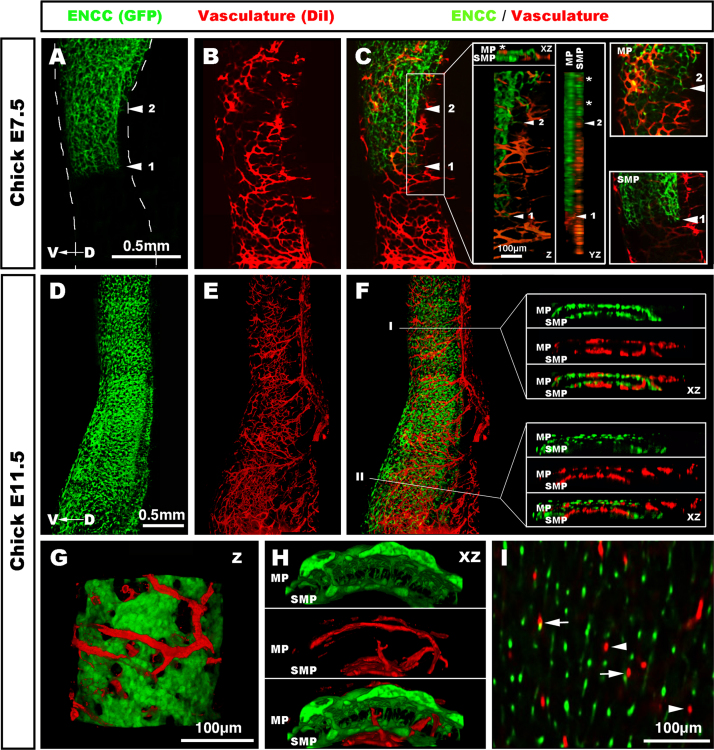
Vascular and enteric network formation in the chick hindgut. (A–C) Confocal imaging of the hindgut at E7.5 (HH32). ENCC are located midway along the hindgut, which already has a dense vascular network. At this stage, the most advanced cells of the migration front (◀1) are present within the presumptive SMP and do not overlap with the vasculature, which is located in the outer layers of the gut (C, insets). XZ and YZ orthogonal views confirm the lack of overlap at the level of the SMP (C, insets). A second group of cells (◀2) is located within the presumptive MP where pre-existing blood vessels are present (C, inset). Despite overlap between blood vessels (^⁎^) and the developing MP, both networks are not systematically juxtaposed (MP inset in C). (D–F) Confocal imaging of the hindgut at E11.5 (HH37-38), the hindgut is fully colonised by both networks. (F, inset I) Transverse sections demonstrate ENCC and blood vessels at both the myenteric and the submucosal levels. (F, inset II) Transverse sections in the most distal part of the hindgut. The presumptive SMP is sparsely populated by ENCC whereas the mucosal vascular supply is very dense. (G and H) High magnification reconstructions reveal a very robust MP intercalated with many blood vessels. The SMP is thinner, and crossed by blood vessels connecting to a dense mucosal blood network medial to the SMP. (I) Image at the level between the MP and the SMP, showing multiple neuronal projections connecting both plexuses. Blood vessels connections are less numerous and are either closely associated with the neuronal projections (arrows) or isolated (arrowheads) with no systematic associations. MP: myenteric plexus; SMP: submucosal plexus. D→V: Dorso-ventral axis. Z: z stack projection 3D reconstruction. XZ: 90° rotation around the *X* axis. Scale bar A–C and D–F=0.5 mm; G and I=100 μm.

**Fig. S3:**
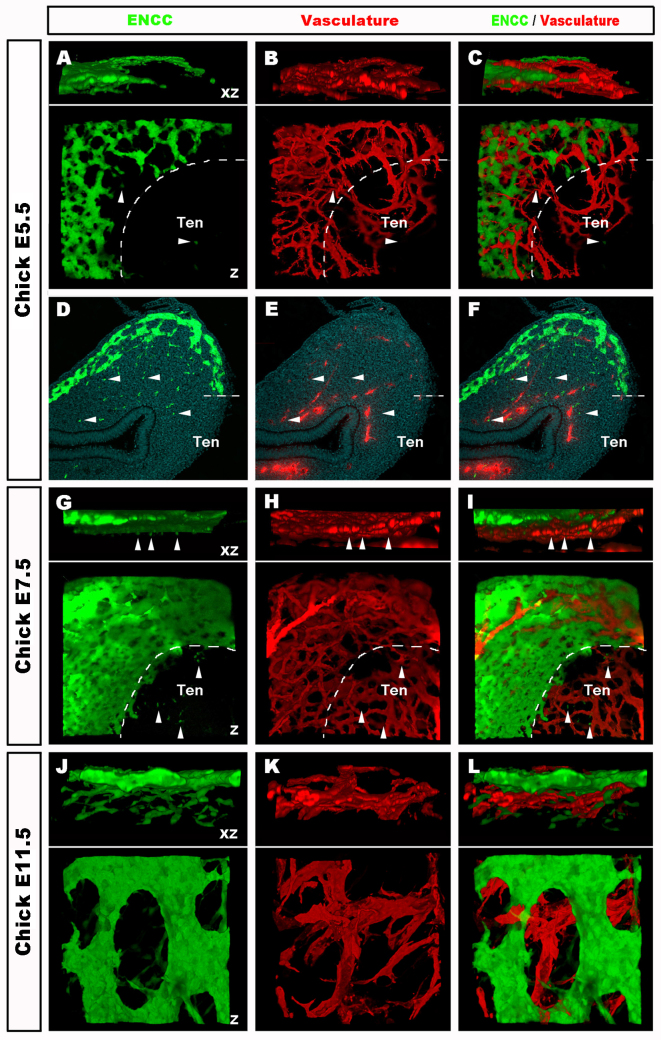
In the embryonic chicken, at the level of the stomach, a rich vascular bed develops underneath a dense peripheral enteric network. (A–C) Images and (D–F) transverse sections of the stomach at E5.5 (HH28), showing a rich vascular bed throughout the tissue, whereas the ENCC network is located more superficially, with the exception of the tendon attachment region where ENCC never migrate (dotted line; Ten). Single ENCC are present deep in the stomach in blood vessel rich regions, although without apparent association with blood vessels (A–F, arrowheads). (G–I) Images of the stomach at E7.5 (HH32) showing a similar pattern as E5.5 (HH28), with denser networks. (J–L) High magnification imaging of the stomach at E11.5 (HH37-38) showing a robust ENS intercalated with large diameter vessels, with the main vascular network located median to the ENS. Z: z stack projection 3D reconstruction. XZ: 90° rotation around the *X* axis.

Along with the stomach (Fig. S3), the hindgut is the only other gut region to be colonised by ENCC after a dense vascular network has already been established (Fig. 2, Fig. 3, Fig. 4). As the ENCC entered the hindgut, they became divided into two separate migration streams. As previously described (Burns and Le Douarin, 1998), the first stream migrated at the level of the presumptive SMP, deep within the tissue and away from the pre-established vasculature (Fig. 4A and C). Close up images, as well as XZ and XY orthogonal projections of the image stacks, confirmed an absence of vasculature at this level (Fig. 4C, insets). The second stream, however, migrated at the level of the presumptive MP, where a pre-established vasculature was present (Fig. 4C, insets). At this level, XZ and XY orthogonal projections clearly demonstrated intercalation of blood vessels (^⁎^) and migrating ENCC (Fig. 4C, insets). Despite this overlap, the migrating paths of the ENCC did not match the orthogonal patterns of the vascular network (Fig. 4C, MP inset). These findings with DiI injection in chick embryos were again confirmed by immunolabeling EC in quail embryos with QH-1. In the quail at E6.5, the migration front of ENCC was located approximately half way along the hindgut (Fig. 3C; arrow). High magnification confocal imaging in the vicinity of the migration front did not show matching patterns between the two networks, despite their close proximity (Fig. 3C; upper box). Further, HNK1+ projections from the nerve of Remak often positioned perpendicular to the QH1+ network (Fig. 3C; lower box).

At E11.5, both networks were fully established throughout the hindgut (Fig. 4E and F; also see Supplementary movie S2). XZ orthogonal projections in the proximal hindgut showed ENCC and blood vessels at both the MP and SMP levels (Fig. 4F, level I). The same projections in the terminal hindgut revealed a sparsely populated presumptive SMP and a very dense vascular network at the level of the mucosa (Fig. 4F, level II). High magnification images and projections demonstrated a very robust MP intercalated with large diameter vessels, spreading laterally around the periphery of the MP (Fig. 4G and H). Z projections at the level of the circular muscle, between the MP and the SMP, showed numerous nerve interconnections (Fig. 4I). In comparison, vascular interconnections at this level were few and did not have systematic associations with the nerve interconnections (Fig. 4I), which again is not supportive of co-dependent developmental interactions.

Supplementary materials related to this article can be found online at doi:10.1016/j.ydbio.2013.11.007.

The following is the Supplementary material related to this article Video 2.

Video 2 Serial Z stack of image in Fig. 3F, showing reconstruction of the hindgut ENS and vascular system at E11.5 (HH37-38).

### Disrupted gut vascular development does not prevent ENCC colonisation of the entire gut

**Fig. 5:**
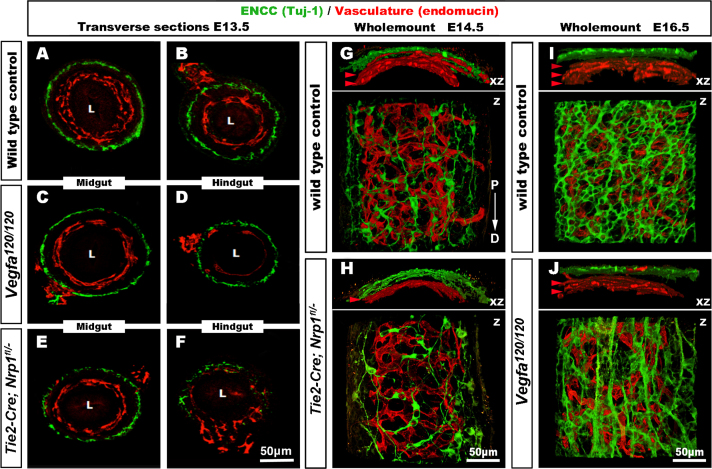
ENCC migrate to the terminal hindgut in *Vegfa*^*120/120*^ and *Tie2-Cre;Nrp1*^*fl*/−^ mutant mice with defective gut vascularisation. (A–F) transverse gut sections from E13.5 mice, labelled with the neuronal marker TuJ1 and the vascular marker endomucin. (G–J) Wholemount preparations of *Tie2-Cre;Nrp1*^*f /*−^ and *Vegfa*^*120/120*^ terminal hindgut at E14.5 and E16.5 respectively. (A and B) Wild type controls have an extensive blood vessel network in the midgut (A), and hindgut (B), with the ENS apparent as an almost continuous ring of TuJ1+ cells encircling the gut. *Vegfa*^*120/120*^ mice have less extensive vascular networks in both the midgut (C) and hindgut (D), but the ENS is similar to controls in both gut regions. (E and F) *Tie2-Cre;Nrp1*^*fl*/−^ mice exhibit more prominent vascular defects, particularly in the hindgut. The ENS is less extensive but ENCC are present within the terminal hindgut. (G–H) At E14.5, controls have an extensive blood vessel network in the hindgut compared to *Tie2-Cre;Nrp1*^*fl/*−^*mice* (red arrowheads). Although less extensive, ENCC have migrated to the terminal end of the gut. (I-J). At E16.5, littermate controls have an extensive blood vessel network in the hindgut compared to *Vegfa*^*120/120*^ mice (red arrowheads). The ENS of *Vegfa*^*120/120*^ mice has a disorganised architecture. Z: z stack projection 3D reconstruction. XZ: 90° rotation around the X axis. P→D: Proximo-distal axis, identical for G–J. Scale bar=50 μm.

To investigate whether ENCC were present in gastrointestinal tracts with disrupted vasculature, we studied *Vegfa*^*120/120*^ mice, which are engineered to express solely the soluble isoform of the essential vascular growth factor VEGF-A and have decreased capillary branch formation in several organs, including the embryonic stomach (Ruhrberg et al., 2002). Whilst their wild type littermates had developed an extensive gut vasculature by E13.5, *Vegfa*^*120/120*^ mutants showed a severe reduction in the hindgut vasculature (Fig. 5). Despite the vascular defects, there were no obvious defects in ENS development in the *Vegfa*^*120/120*^ mice (Fig. 5A–D). At E16.5, the reduction of the gut vasculature was obvious in *Vegfa*^*120/120*^ mice compared to controls (Fig. 5I and J). Although very disorganised in its architecture, the ENS of *Vegfa*^*120/120*^ mice was similar to controls in that enteric neurons were present along the entire length of the gut (Fig. 5I and J). Further, TuJ1+ cells counts in equivalent regions of the hindgut were not significantly different (data not shown). We also examined *Tie2-Cre;Nrp1*^*fl/*−^ mice, in which NRP1 is conditionally knocked out in vascular endothelial cells (Fantin et al., 2013, Gu et al., 2003) The vascular defects in the *Tie2-Cre;Nrp1*^*fl/*−^ hindgut were more severe than in *Vegfa*^*120/120*^ mice (Fig. 5E–H). Despite the severity of the vascular phenotype, ENCC were present within the terminal hindgut at E13.5 (Fig. 5E and F) and at E14.5 (Fig. 5G and H), indicating that their migration along the gut was not delayed. Cell counts of TuJ1+ cells in equivalent hindgut regions showed a statistically significant (*p*=0.035) reduction in TuJ1+ ENS cells in the mutants (mutants 361±15, *n*=7 *versus* controls 439±28, *n*=8).

### ENCC colonise the entire length of the hindgut in the absence of vasculature

**Fig. 6:**
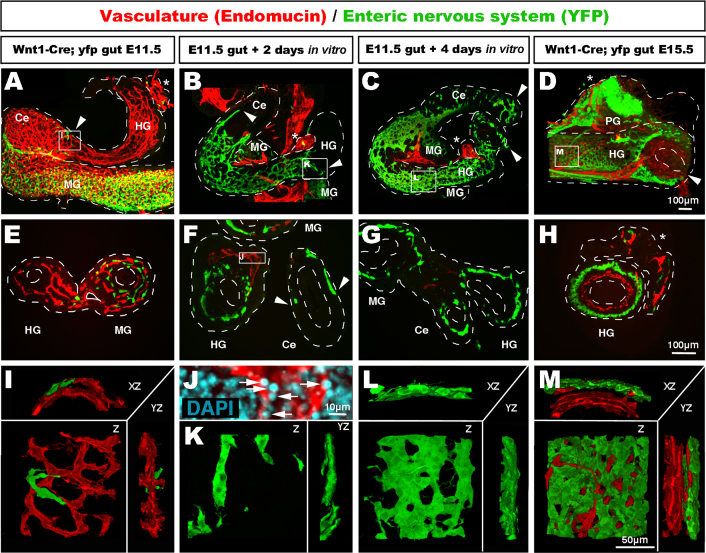
ENCC colonise the entire length of the mouse embryo hindgut *in vitro* in the absence of a vascular network. (A–D) Wholemount and (E–H) transverse sections through *Wnt1-Cre;R26R*^*yfp/+*^ mouse gut labelled with endomucin (vasculature) and YFP (ENCC). (A, E, and I) At E11.5, only occasional ENCC are present in the hindgut (A, arrowhead, close up in I). E11.5 guts were dissected and placed in culture for up to 4 days. (B, F, J, and K) Confocal images showing guts after 2 days and (C, G, and L) after 4 days of culture. The vascular network collapse is visible by reduced endomucin staining at 2 days (B and F) and 4 days (C and G), and by the presence of apoptotic rounded nucleus in endomucin+ tissue (J, arrows, inset from F). Despite the collapse of the vascular network, ENCC fully colonise the entire length of the hindgut, as seen by YFP staining (B, C, F, and G, arrowheads). Chain migrating ENCC occur with and without the presence of a vascular network (B, F, and K). (C) Wholemount (G) section and (L) high magnification confocal imaging show that, after 4 days in culture, ENCC form a circular plexus comparable with E15.5 non-cultured controls (D, H, and M). MG: midgut; Ce: caecum; HG: hindgut; PG: pelvic ganglion. ^⁎^Inferior mesenteric supply. Z: z stack projection 3D reconstruction. XZ/YZ: 90° rotation around the *X* or *Y* axis. Scale bar A–D and E–H=100 μm; I–M=50 μm.

**Fig. S4:**
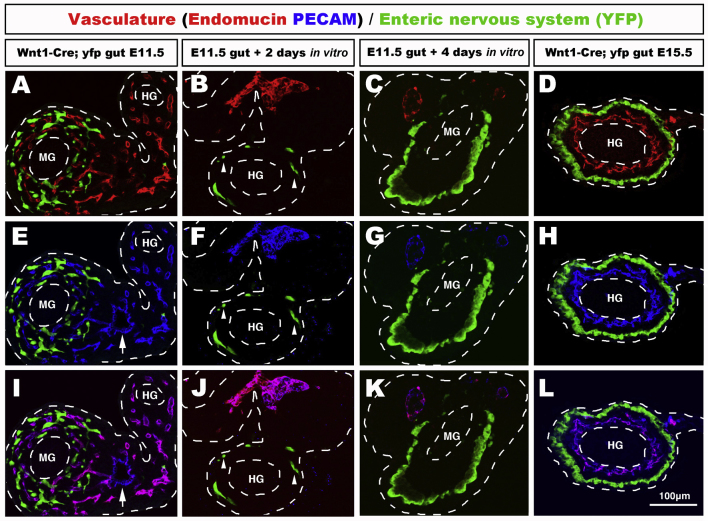
ENCC colonise the entire length of the embryonic mouse hindgut *in vitro*, in the absence of PECAM and endomucin+ cells. (A–L) Transverse sections through *Wnt1-Cre;R26R*^*yfp/+*^ labelled with endomucin (vasculature) and YFP (ENCC). At E11.5, the superior mesenteric artery is PECAM+ and endomucin −ve (A, E, and I; arrow). During *in vitro* culture, the vascular network collapses due to the lack of blood flow, as seen by reduced endomucin and PECAM staining at 2 days (B and F) and 4 days (C and G). Despite the absence of PECAM and endomucin+ cells in the gut, ENCC fully colonise the entire length of the hindgut, as seen by YFP staining (B, C, F, and G, arrowheads). Chain migrating ENCC occur with and without the presence of PECAM and endomucin cells (B, F, and K). MG: midgut; HG: hindgut.

Next we took advantage of the fact that, in organ culture *in vitro*, the vascular system collapses and degenerates due to the lack of blood flow, to determine whether ENCC could migrate along the hindgut in the absence of a vascular system. We used *Wnt1-Cre;Rosa26*^*Yfp/+*^ mice, which express YFP in NCC (Jiang et al., 2000), including ENCC (Druckenbrod and Epstein, 2005). Wholemount staining of E11.5 *Wnt1-Cre;Rosa26*^*Yfp/+*^ midgut and hindgut with endomucin and YFP showed that the hindgut was largely free of ENCC at this stage, but contained a pre-established vascular network (Fig. 6A, E and I). High magnification views of the hindgut at E11.5 showed that the few ENCC present migrated in the vicinity of, yet separate from, the vasculature (Fig. 6I). We then cultured the E11.5 guts for up to 4 days. Endomucin and PECAM staining showed that the vascular network within the gut tube had disappeared after two days in culture, and only remnants of the mesentery were present adjacent and exterior to the gut wall (Fig. 6B, F and K; Fig. S4). In particular, the remnants of the inferior mesenteric supply were a noticeable landmark for the terminal part of the hindgut (Fig. 6A–D and H). Numerous condensed apoptotic nuclei in endomucin+ cells demonstrated the collapse of the vascular system (Fig. 6J). Despite the lack of a vascular network within the gut, ENCC still migrated within the caecum and hindgut (Fig. 6B, F and K; Fig. S4). High magnification images in the terminal hindgut showed ENCC undergoing chain migration in the absence of endomucin+ cells (Fig. 6K). After four days in culture, the collapse of the vascular system led to the disappearance of most of the mesentery, while the colonisation by ENCC carried on to the terminal ends of both the caecum and hindgut (Fig. 6C and G; Fig. S4). At this stage the ENCC formed an ENS in the hindgut, which was less dense than that of E15.5 controls (Fig. 6C, D, G, H, L, and M). Thus the total lack of a vascular system did not affect the ability of ENCC to colonise the entire length of the hindgut and organise into an ENS.

### The hindgut enteric vasculature develops normally in the absence of the ENS

**Fig. 7:**
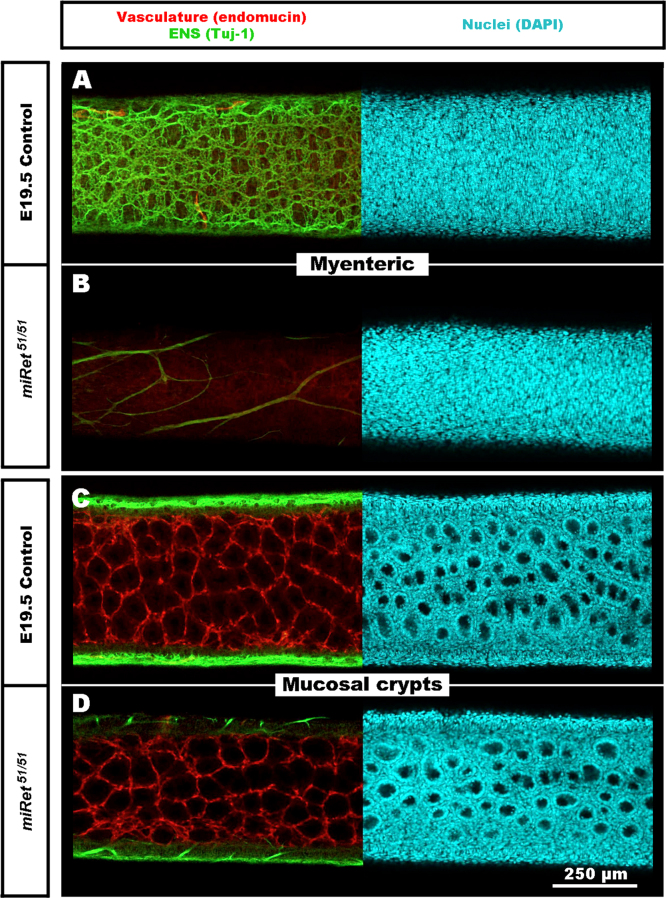
The enteric vascular system appears normal in the aganglionic terminal hindgut of *miRet*^*51*^ mutant mice at E19.5. (A–D) wholemount preparations of the terminal hindgut of *miRet*^*51*^ and control littermates at E19.5 labelled with the neuronal marker TuJ1, the vascular marker endomucin and the nuclear marker DAPI. (A and B) confocal images at the level of the myenteric plexus reveal an extensive ENS in control littermates (A) whereas in *miRet*^*51*^ mice (B) only sparse extrinsic nerves are present. (C–D) Confocal images at the level of the mucosal crypts demonstrate similar vascular networks in control littermates and *miRet*^*51*^ mice. Scale bar=250 μm.

Finally, to investigate whether the vasculature develops normally in the absence of ENS, we examined monoisoformic *miRet*^*51*^ mice, which lack ENS in the hindgut and are an established mouse model of Hirschsprung disease (de Graaff et al., 2001). At E19.5, a stage at which both the ENS and vasculature are normally well established, wild type littermates had an extensive ENS in the hindgut, whereas *miRet*^*51*^ mutants lacked an ENS, with only occasional extrinsic nerve fibres present in the terminal hindgut (Fig. 7A and B; Supplementary movie S3). Imaging of the vascular bed at the level of the mucosal crypts showed no obvious difference in the vascular networks between wild type and *miRet*^*51*^ mutants (Fig. 7C and D; Supplementary movie S3). These findings suggest that vascular development in the hindgut occurs independently from the ENS.

Supplementary materials related to this article can be found online at doi:10.1016/j.ydbio.2013.11.007.

The following is the Supplementary material related to this article Video 3.

Video 3 Serial Z stack of confocal images showing reconstruction of the hindgut ENS (TuJ1), vascular system (endomucin) and tissue histology (DAPI) at E19.5 in control littermates (upper panel) and *miRet*^*51*^ mutant mice (lower panel), as shown in F

## Discussion

Here we investigated the interrelationship between migrating ENCC and the developing vasculature of the gut. In avian models we found that both networks displayed very different early developmental patterns: ENCC migrated in areas devoid of vasculature, and in areas where the vasculature was present, they frequently migrated without closely associating with existing blood vessels. These results suggest that the presence of a vascular network is not necessary for ENCC to colonise the digestive tract. To corroborate these findings, we looked at two different strains of mutant mice where the gut vasculature is defective and found that ENCC migrated along the entire length of the gut and formed an ENS despite the presence of severe vascular defects. We also tested the migratory ability of ENCC in the complete absence of vasculature, using an *in vitro* gut culture system and found, once again, that ENCC were able to complete their migration and form an ENS along the entire hindgut. Finally, using a mouse model of Hirschsprung disease that lacks hindgut ENS, we showed that early vascular patterning was unaffected by the absence of an ENS.

In this study we used the well established technique of neural tube transplantation to label ENCC (Burns and Le Douarin, 2001, Freem et al., 2012). We selectively labelled vagal NCC, as the sacral NCC that colonise the hindgut have previously been reported to use extrinsic nerves projections as their migratory route into the gut (Burns and Le Douarin, 1998, Erickson et al., 2012, Wang et al., 2011), likely ruling out a direct role for mesenteric vasculature in their guidance. Using a vagal neural crest transplant also allowed us to label NCC migrating to and within the respiratory system, as intrinsic lung neurons derive from the same region of the neuraxis as ENCC (Burns and Delalande, 2005, Freem et al., 2012, Freem et al., 2010). Because of their function in gas exchange, the lungs develop an extremely dense capillary network. If the vasculature were essential for guiding NCC, one would expect that NCC would be intimately associated with these blood vessels. However, we observed the contrary: streams of migrating NCC initially colonised the medio-dorsal aspect of the lungs, whereas the nascent capillary network was located on the lateral side. Thus the observations in the respiratory system are consistent with our observations in the digestive tract, with neither organ highlighting a role for the vasculature in NCC migration.

Overall, our results make sense from an evolutionary point of view, since the nervous system is already present in invertebrates such as nematodes (which lack a vascular system and diffuse oxygen through tracheoles) suggesting that its development is based on an ancient developmental programme, predating and independent of vascular development (Eichmann and Thomas, 2013, Munoz-Chapuli, 2011, Popovici et al., 2002). Our results are also in accordance with previous studies showing that the neurovascular congruence observed during limb development results from shared patterning mechanisms and not co-dependent developmental programs (Bates et al., 2003, Bates et al., 2002). In the 2003 study, ablation of forelimb-level neural tube to produce aneural limbs resulted in a completely normal vascular network. Conversely, alteration of the vascular pattern using beads soaked with VEGF_165_, VEGF_121_, Ang-1 or Flt-1/Fc resulted in normal peripheral nerve patterning. Bates and colleagues concluded that neurovascular congruency was determined by shared molecular pathways, including Sema3A and NRP1, guiding both developing networks in the vicinity of each other. Our results are consistent with a similar model in the digestive tract where shared mechanisms between neural and vascular development have also been demonstrated. In particular, β-1 integrins have been shown to be required cell autonomously for the migration of both EC and ENCC (Breau et al., 2009, Broders-Bondon et al., 2012, Carlson et al., 2008). This model is further supported by the fact that extracellular matrix components (*e.g.* laminin, tenascin and collagens), regulating migration of both NCC and EC, are present only in discrete gut territories, potentially restricting the colonisation path of both networks and enforcing their proximity in some areas and not others (Breau et al., 2009, Broders-Bondon et al., 2012, Herbst et al., 1988, Nagy et al., 2009, Zagzag et al., 2002).

Our results, however, are in contradiction with a previous study from Nagy and colleagues, looking at the migration of ENCC in the hindgut of quail embryos, which suggested an essential role for the microvasculature in ENS formation by guiding ENCC migration via β-1 integrin signalling (Nagy et al., 2009). A possible reason for this contradiction is that the β-1 integrin requirement for the migration of both ENCC and EC had not been demonstrated at the time of Nagy and colleagues' publication. Additionally, technical differences may also contribute to these conflicting results. Importantly, classical EC markers, such as endomucin, PECAM or the quail specific marker QH1, do not work in chicken. Instead, we used DiI for vessel painting, which allowed us to stain the entire lumenised network. This technique allowed us to examine the relationship between ENCC and patent blood vessels, but did not identify unopened capillaries, endothelial tip cells or isolated endothelial cells. We therefore used the quail embryo as a second avian model, because of the availability of QH1 antibody to label endothelial cells, to confirm and extend our findings in the chick. In addition, the second part of our study was performed using mouse tissues, in which all EC can be readily immunolabeled with endomucin and PECAM. Overall, the results were consistent with our observations in chicken embryos, giving us confidence in the validity of the vascular labelling technique. Nagy and colleagues also only looked at the colonisation of the terminal hindgut, where vasculature develops prior to ENCC migration. Our study covered the development of the entire digestive tract and clearly demonstrated examples of regions, such as the oesophagus, where ENS development precedes vascularisation. We also show that, in regions such as the midgut, migration of the ENCC happened in a direction perpendicular to the blood vessel layout, which contradicted the idea that blood vessels were guiding the migration. In the regions were both networks developed in very close proximity, there was no evidence of congruence, as previously reported (Landman et al., 2011, Young et al., 2004).

Despite the differences between our findings and those of Nagy et al., both studies clearly document examples of close associations between the developing vascular network and migrating ENCC. This suggests that blood vessels, although not necessary for ENCC migration, may provide a suitable substrate for their migration. Indeed, the basement membrane of EC has been shown to express all the required extracellular matrix components to support NCC migration (Bou-Gharios et al., 2004) and can therefore be used as a migratory pathway when vessels are laid out prior to the arrival of NCC. Our results, however, demonstrate that this association is not systematic or even required for ENCC migration. Conversely, we found that the early development of the enteric vascular system was unaffected by aganglionosis. These results again support the idea that the early developmental programs of both networks are independent from each other. However, they are sometimes brought together in close proximity during early development, perhaps because they rely on similar molecular or cellular mechanisms to colonise their target organs.

Recently an important role for the interaction of the vasculature and migrating ENCC was suggested by the observation that a sub-population of ENCC, termed trans-mesenteric ENCC (tmENCC), migrate through the gut mesentery as a shortcut to reach the terminal hindgut, and that these cells represent the principal source for ENS formation in the colon of mice (Nishiyama et al., 2012). Strikingly, tmENCC break away from the main migrating streams within the distal midgut and progress as individual cells through the mesenteric vasculature to reach the terminal hindgut ahead of the main migration front. Despite the presence of ENCC in the mesentery (data not shown), there is no published evidence for tmENCC in chicken. Nevertheless, our study in the chicken embryo also shows examples of isolated NCC migration away from the main migration stream and towards vascular rich areas in the lungs and in the stomach. These results raise the possibility that the vasculature triggers this “lone” migrating behaviour via secreted molecules. Artemin, a neurotrophic factor expressed by blood vessels, is a possible candidate to account for this (Honma et al., 2002). Another possible candidate is the SDF1 chemokine pathway, although Nishiyama and colleagues showed by *in situ* hybridisation that tmENCC do not express *cxcr4*, one of the chemokine receptors for SDF1 (Nishiyama et al., 2012).

Our results using *VEGF*^*120/120*^ and *Tie2-Cre;Nrp1*^*fl/−*^ mice demonstrated the ability of ENCC to colonise the entire length of the hindgut and form the ENS, despite severe blood vessels defects. Although the ENCC were able to migrate all the way to the terminal hindgut, the resulting ENS was disorganised and sparse compared with wild type controls. In the case of the *Tie2-Cre;Nrp1*^*fl/*−^ mice, we observed a statistically significant reduction in the number of ENS cells. Likewise, our results in chick suggest a connection between the density of the ENS and that of the vascular bed, with the oesophageal ENS being sparser than that of the stomach or hindgut, which are both richly vascularised. The interpretation of such results is, however, complicated by the fact that vascular insufficiency not only reduces the ability of the ENCC to interact with EC, but also has indirect effects due to impaired oxygen and nutrient supply. For that reason, *in vitro* experiments, where nutrients are provided artificially, are a valuable experimental tool. Here we used gut explant culture, which interrupts blood flow to the explant from the moment it is cultured. It has been well documented that interruption of the blood flow leads to the collapse of the capillaries and a catastrophic induction of apoptosis in EC, both *in vivo* and *in vitro* (Jenkins et al., 2013, Meeson et al., 1996, Meeson et al., 1999). We took advantage of this vasculature collapse (assessed by the disappearance of both Endomucin+ and PECAM+ cells within the intestinal tissues) to demonstrate the ability of ENCC to migrate and form an ENS in the absence of endothelial cells and/or the vascular system. Nishiyama and colleagues also used gut explant culture and although they showed a delay in the advance of ENCC along the hindgut when the mesentery was removed, in accordance with our results, the gut explants were eventually fully colonized (Nishiyama et al., 2012). Nevertheless, it is important to point out that our results do not rule out the possibility that a vascular defect could lead to ENS defects by reducing the number of tmENCC and/or the failure to meet the metabolic demands of migrating ENCC during critical developmental windows. Since our studies mainly utilised neural crest markers such as chick-GFP and Wnt1-Cre, they did not shed light on the subsequent survival of NCC derivatives or different lineages within the ganglia and/or other abnormalities of ENS structure or function that may arise later in development due to decreased vascularisation.

In conclusion, our results show that blood vessels are not necessary for guiding migrating ENCC during ENS development. However, there may be interactions between ENCC and endothelial cells prior to the establishment of patent blood vessels, and/or the establishment of functional neuro-vascular units in the gut later in development, as the ENS is subtly altered when vascularisation is reduced. Recent advances in imaging techniques and “optical clearing” of gut tissues now make it possible to investigate later developmental stages at which the gut neuro-vascular units likely become established. These, and further studies in mice deficient in ENS or in the gut vasculature, will shed light on the shared developmental mechanisms that underlie normal gut neurovascularisation.

## Footnotes

Appendix A Supplementary data associated with this article can be found in the online version at doi:10.1016/j.ydbio.2013.11.007.
