# Supplementary material for: Vascularisation is not necessary for gut colonisation by enteric neural crest cells
Source: Dev Biol. 2014 Jan 15;385(2):220–9. doi: 10.1016/j.ydbio.2013.11.007 (PMC3928993; doi:10.1016/j.ydbio.2013.11.007)
Supplement: Supplementary file 4 — Supplementary data [file mmc8.doc]

**Supplementary data:**

Tie2Cre:

5'-TGCCACGACCAAGTGACAGCAATG-3'

5'-ACCAGAGACGGAAATCCATCGCTC-3'

NRP1:

5'-AGGCCAATCAAAGTCCTGAAAGACAGTCCC-3'

5'-AAACCCCCTCAATTGATGTTAACACAGCCC-3'

VEGF120:

5'-CAGTCTATTGCCTCCTGACCTTCAGGGTC-3'

5'-CTTGCGTCCACACCGTCACATTAAGTCAC-3'

5'-TTCAGAGCGGAGAAAGCATTT GTTTGTCCA-3'
